# Image analysis-derived metrics of histomorphological complexity predicts prognosis and treatment response in stage II-III colon cancer

**DOI:** 10.1038/srep36149

**Published:** 2016-11-02

**Authors:** Artur Mezheyeuski, Ina Hrynchyk, Mia Karlberg, Anna Portyanko, Lars Egevad, Peter Ragnhammar, David Edler, Bengt Glimelius, Arne Östman

**Affiliations:** 1 Department of Oncology-Pathology, Karolinska Institutet, Stockholm, Sweden; 2 Department of Pathology, Belarusian State Medical University, Minsk, Belarus; 3 City Clinical Pathologoanatomic Bureau, Minsk, Belarus; 4 Department of Molecular Medicine and Surgery, Karolinska University Hospital Solna, Stockholm, Sweden; 5 Department of Immunology, Genetics and Pathology, Uppsala University, Uppsala, Sweden

## Abstract

The complexity of tumor histomorphology reflects underlying tumor biology impacting on natural course and response to treatment. This study presents a method of computer-aided analysis of tissue sections, relying on multifractal (MF) analyses, of cytokeratin-stained tumor sections which quantitatively evaluates of the morphological complexity of the tumor-stroma interface. This approach was applied to colon cancer collection, from an adjuvant treatment randomized study. Metrics obtained with the method acted as independent markers for natural course of the disease, and for benefit of adjuvant treatment. Comparative analyses demonstrated that MF metrics out-performed standard histomorphological features such as tumor grade, budding and configuration of invasive front. Notably, the MF analyses-derived “**α**_**max**_” –metric constitutes the first response-predictive biomarker in stage II-III colon cancer showing significant interactions with treatment in analyses using a randomized trial-derived study population. Based on these results the method appears as an attractive and easy-to-implement tool for biomarker identification.

Adjuvant treatment of stage II-III colon cancer has significantly improved outcome and is now widely implemented based on a series of positive randomized trials. A major challenge in this field is a better definition of high and low risk groups, differing in their need for adjuvant treatment^1^,^2^.

Routine adjuvant chemotherapy is not recommended for stage II colon cancer. However, a number of high-risk patients in stage II group could benefit from adjuvant treatment. The high-risk criteria according to ASCO recommendations include number of sampled lymph nodes, low tumor differentiation and perforation of T4 stage^3^. ESMO criteria also include vascular invasion, lymphatic or perineural invasion, obstruction and high levels of carcinoembryonic antigen^4^.

The recommendations for stage III colon cancer are more uniform and suggest routine usage of adjuvant chemotherapy. However, it is well recognized that the stage III population, in addition to adjuvant therapy-responding patients, also include treatment-refractory cases.

Taken together, this suggests that better criteria are needed for accurate definition of patients who would benefit from adjuvant treatment in both stage II and stage III colon cancer. Due to improvements in the general care of patients with colon cancer, resulting in fewer recurrences, and an associated stage migration, the routine use of adjuvant chemotherapy in several patient groups has been challenged. Much more elaborated stratification is needed for selection of patients needed to treat^5^. There is presently no good knowledge about the risk of recurrence in adequately staged, operated and pathologically examined patients^6^.

The histopathological organization of colon tumor tissue is routinely evaluated as the grade of tumor differentiation. Tumor-stroma interactions and architecture of the invasive border are evaluated by defining tumor border configuration (TBC) (pushing, intermediate and infiltrative) and grade of budding (TB). These scores are related to the prognosis, and are applied or recommended for the pathological reports^7^,^8^,^9^. However, none of these histomorphological markers predict response to treatment. Notably, scoring of grade, TBC and TB is subjective, not standardized and may be time-consuming.

Digital image analysis of slide section scans allows quantitative and observer-independent characterization of morphological and histomorphological features. Unsupervised and supervised analyses of tumor morphology has identified novel prognostic markers and identified metastasis-associated patterns of immune infiltration^10^,^11^. The complexity of the tumor structure can be also described by fractal geometry. A few studies have applied multifractal (MF) analyses to breast^12^, colorectal^13^,^14^, prostate^15^ and lung tumor collections (reviewed in ref. 16). Some of these studies have identified associations between specific MF metrics and outcome. However, none of the earlier studies have analyzed material from randomized studies, which have prevented clear distinctions between marker impact on natural course/prognosis and response to treatment.

In this study we have analyzed prognostic and response-predictive capacity of two MF metrics collected by digital image analyses of cytokeratin-stained sections from 291 tumors of stage II-III colon cancer patients participating in an earlier reported randomized trial comparing treatment with surgery alone and surgery and adjuvant fluoropyrimidine chemotherapy^17^. Scores for grade, TB and TBC were also collected from the same cases and related to MF metrics and outcome.

## Material and Methods

### Patients

The 291 patients were derived from a randomized clinical Nordic trial aiming to evaluate the efficacy of 5-FU (5-fluorouracil) based adjuvant chemotherapy^17^. The original study included 2224 patients under the age of 76 year with radically resected stage II and III colorectal cancer during the time period 1991–1997. The patients were randomized to surgery alone or surgery followed by 5-FU based adjuvant chemotherapy. The adjuvant chemotherapy regimens included 5FU/leucovorin for 4–5 months according to either a modified Mayo Clinic schedule or the Nordic schedule or 5FU/levamisole for 12 months. Some centers also randomized patients treated with 5FU/leucovorin to +/− levamisole. This study is based on a selection of Swedish cases from the original study. None of the patients were treated with radiotherapy or chemotherapy prior to surgery. Clinical data on patients and tumor characteristics were retrieved through pathology reports. Survival data were available through the regional centers of epidemiological oncology. Patient characteristics are listed in Table 1.

All methods were carried out in accordance with relevant guidelines and regulations. All participants have given and written informed consent, which also included prognosis-related studies. The ethical committee of the Karolinska Institutet, Stockholm, Sweden, approved the analysis within this cohort (Dnr 00–260, 2014/664–32).

### IHC procedures

A pathologist reviewed available tumor sections stained with H&E. One section per case with the area of deepest tumor invasion was selected. 4 μm thick sections were cut from the selected tissue blocks and mounted on slides. Sections were de-paraffinized and rehydrated in ethanol. Antigen retrieval was carried out using citrate buffer (pH 6.0) in Pascal Pressure Cooker or microwave oven. Endogenous peroxidase was blocked with 3% H2O2 (20 min). The sections were incubated at +4 °C overnight with the monoclonal mouse pan-cytokeratin antibody MNF-116 (DakoCytomation, Denmark), 1:75, followed by incubation with the amplification system (EnVision + System-HRP Dako A/S, EnVisionTM, Dako, CA, USA) for 30 min at room temperature. Diaminobenzidine (DAB) detection system was used for specific staining and hematoxylin as counterstain.

### Slide digitalization

Slides were scanned by Vslide slide scanning microscope (Metasystems, Alltlussheim, Germany) with ×10 objective and RGB led illumination for color deconvolution. The program Metaviewer (Metasysetms, Alltlussheim, Germany) was used to view the scanned digital slides and to save them in.tif format at a resolution 0.65 micrometers per pixel.

### Evaluation of TBC and TB

TBC was assessed according to recommendations of Morikawa *et al.*^18^. In short, the growth pattern was evaluated at low magnification and categorized as pushing (expansile), intermediate, or infiltrative. Well circumscribed growth pattern considered as pushing. The appearance of irregular clusters, cords of cancer cells or small glands without distinct border considered as infiltrative pattern. Intermediate growth pattern was associated with irregularity of the large and medium-sized glands at the invasive border.

TB assessment was done according to the guidelines published by Karamitopolou *et al.* 2012^19^. The average number of tumor buds in 10 fields of view was then used either as continuous value (for correlation analyses) or was used to dichotomize cases into high-budding group (≥10 per high-power field area) and low-budding group (<10 buds).

The information about the grade of the tumor differentiation was retrieved from the clinic-pathological records.

### Digital image analysis

#### Inclusion criteria and area selection

For the digital analyses a subset of 291 cases was used. The major exclusion criteria were partial tissue detachment from the glass, focusing issues of the slide scanning microscope which resulted on blurred digital image of >10% of sample area or poor quality of immunohistochemical staining (too weak specific staining and/or too strong background staining).

For quantification of MF metrics one region per case was selected. All areas selection was done blinded to out-come data. Selection of areas for analyses was based on the following criteria:

1. The largest possible tissue region was selected if not contradictory with other selection criteria.
2. The selected area should include stroma/tumor interface regions.
3. Areas of artificial tissue damage should be avoided.
4. When more than one tumor tissue sample/slide was available from each case, analyses were based on the sample with the deepest invasion.

#### Image segmentation and classification

The intention was to get as large tumor area as possible, excluding sample margins and areas with tissue damage or unspecific staining. For the classification of tumor and non-tumor regions we used Definiens eCognition Developer^®^ software trial version 9.1. The analytical pipeline consisted of the segmentation of the image, derived from the pan-cytokeratin-stained section (Fig. S1A), and subsequent image classification using nearest neighbour object-based algorithm. The pipeline was developed and tuned on 10 randomly selected images, and then applied for the rest of the samples. The regions of the tumor tissue, tumor stroma and “background areas” were classified. Each image was reevaluated by pathologist (AM), and classifier settings were modified if deemed necessary. The classified images were then binarized into “tumor vs. non-tumor” images (Fig. S1B). The first type of images was made by outlining of cancer contour, and thus represented tumor-stroma interface (Fig. S1C1). The second type of images, in addition to the tumor-stroma outlines, also contained contours of internal structures of the tumor tissue (Fig. S1C2).

#### Multifractal analysis

**Figure 1:**
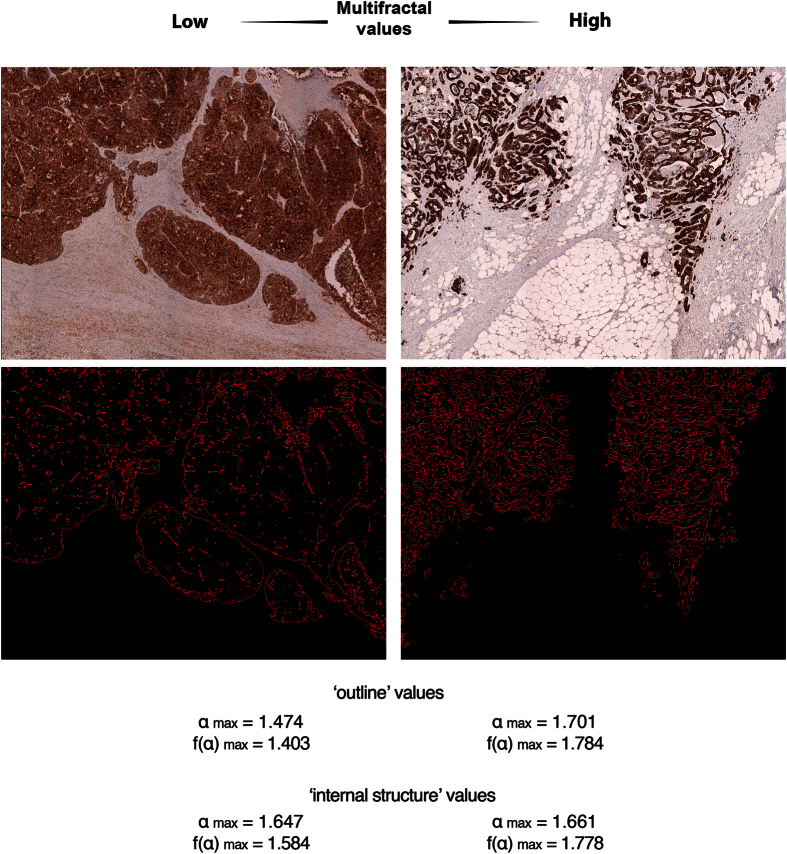
Representative images from cases with low and high MF metrics scores. Two tumors are shown after pan-cytokeratin staining and after completion of “contour-formation”. Cases are selected to illustrate low and high MF metrics scores. **α**_**max**_ and ***f*****(α)**_**max**_ scores are shown as for ‘outline’-based and ‘internal structure’-bases image analyses.

The multifractal analysis was performed on contour images, obtained as described above, using the FracLac plug-in for the freely available ImageJ software (Karperien, A., FracLac for ImageJ. http://rsb.info.nih.gov/ij/plugins/fraclac/FLHelp/Introduction.htm. 1999–2013.) FracLac default settings were used (the box sizes (scale window) were set for the range from 10 pixels (min) to the maximal of 60% of an image, Q range from −10 to +10; no multifractal filters were used; minimal density 0.1 and maximal density 0.98). The generalized fractal dimension *D*(*q*) *vs Q* spectra was generated for each case and the correlation coefficient (r^2^) for the regression line for the *D*(*q*) *vs Q* was computed. The Hölder’s exponent (‘**α**’) reflects the local regularity of the analyzed structure. High values of **α** indicate high local irregularity of the observed structure. For the analyses in the current study we utilized maximal scores for *local irregularity* among the scores achieved on different scales - **α**_**max**_. The second metric is produced as function of **α** making multifractal spectrum - ***f*****(α)** – and characterizes the *global irregularity*. FracLac-derived data were used to obtain, for each tumor, four multifractal metrics: **α**_**max**_ and ***f*****(α)**_**max**_ for the ‘outline’ images (Fig. S1D1) and **α**_**max**_^**internal structure**^ and ***f*****(α)**_**max**_^**internal structure**^ for the images with internal structures (Fig. S1D2). The representative images of two tumor samples from the analyzed cohort with low and high multifractal metrics are shown in Fig. 1.

### Statistical analysis

Spearman’s two tailed test was used to test correlation between continuous variables.

For the comparisons of the continuous variables in clinico-pathological subgroups, Mann Withney and Kruskal Wallis tests were used.

For survival analyses Cox Regression was performed in uni- and multivariate settings with median-based dichotomization of cases into “metric-high” and “metric-low” groups.

All statistical tests were performed using SPSS V20 (SPSS Inc., Chicago, IL) and R (version 3.2.2). p values < 0.05 were considered statistically significant.

As survival endpoints we used Cancer Specific Survival (CSS), which was measured from the date of surgery to the date of death from CRC, and Time To Recurrence (TTR), which is time from the date of surgery to the date of local recurrence, distant metastases or death from CRC.

To estimate interactions between adjuvant treatment and studied factors, the test of interaction was used, and included analyzed characteristic (high/low), adjuvant chemotherapy (+/−) and a product them (analyzed characteristic * adjuvant chemotherapy).

## Results

### Multifractal properties of the dataset

Scaling selection has been shown to be critical for the multifractal (MF) analyses^20^,^21^. To show that the parameters for the MF analysis in our study are selected properly we performed a series of analyses to investigate the behavior of the data set. The individual data from the spectra of the generalized fractal dimension *D*(*q*) of each sample was used to produce a summary plot of *D*(*q*) *vs Q* spectra for the whole cohort. As shown in Supp Fig. S1E, the *D*(*q*) *vs Q* curve is nonlinear, has sigmoid shape and is descending. This indicates that the analyzed data has MF properties within the selected settings and thus can be subjected for MF analysis^22^. The average correlation coefficient (r^2^) for the regression lines for the *D*(*q*) *vs Q* was equal to 0.826 (standard deviation 0.063), which indicates good fit of the line to the data.

### Study population and multifractal metrics collection

Clinico-pathological characteristics of the study population are described in Tables 1 and 2 and in Tables S1 and S2.

The mean CSS in the subgroup treated with surgery alone (n = 150) was 96 months compared with 102 months in patients receiving adjuvant chemotherapy (n = 141). This difference was not statistically significant (Fig. S2). The mean TTR was 90 and 95 months for the surgery-alone and the adjuvant group, respectively, with no statistically significant difference between the groups (Fig. S2).

Digital image analysis of cytokeratin-stained samples was used to collect data from each case for four MF metrics: **α**_**max**_, ***f*****(α)**_**max**_, **α**_**max**_^**internal structure**^ and ***f*****(α)**_**max**_^**internal structure**^ (see Materials and Methods for details) (Fig. 1).

MF metrics displayed only moderate associations between each other with Spearman correlation coefficients r = 0.650 (for **α**_**max**_ and ***f*****(α)**_**max**_) and r = 0.547 (for **α**_**max**_^**internal structure**^ and ***f*****(α)**_**max**_^**internal structure**^) (p < 0.001).

### Association of MF metrics with clinicopathological and histomorphological characteristics

As shown in Table 1, proximal localization was associated with higher score for both multifractal metrics. Furthermore, both digital metrics were higher in tumors from females than males. No associations were found with patient age, DNA mismatch repair (MMR) status or usage of adjuvant chemotherapy.

Analyses of correlations between the two MF metrics and histomorphological features demonstrated that both MF metrics were associated with TB and TBC (Table 2). Only ***f*****(α)**_**max**_ was associated with tumor differentiation.

Analyses which included internal tumor structures are available in Supp. Tables 1 and 2. Interestingly, among clinicopathological characteristics only tumor localization was associated with **α**_**max**_^**internal structure**^ and ***f*****(α)**_**max**_^**internal structure**^.

### Multifractal parameters are associated with the post-surgery natural course of stage II-III colon cancer

A set of analyses was performed with the aim to analyze if the MF metrics were associated with the intrinsic aggressiveness of the disease. For this purpose the associations between MF metrics and TTR were analyzed in the subgroup of patients not receiving adjuvant chemotherapy.

**Figure 2:**
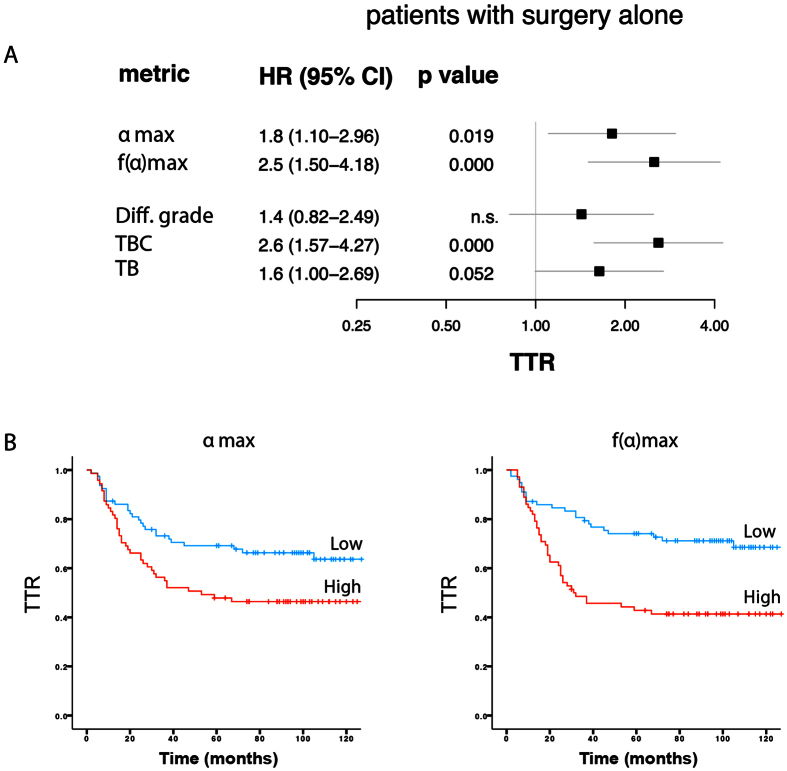
Survival-associations for MF metrics and histomorphological scores in stage II-III colon cancer treated with surgery alone. (**A**) Association between MF metrics (**α**_**max**_ and ***f*****(α)**_**max**_), histomorphological features and TTR. Cox regression analyses were used for determination of HRs. (**B**) Kaplan-Meier plots illustrating TTR of stage II-III colon cancer treated with surgery alone after median-based dichotomization of cases based on the ***f*****(α)**_**max**_ metric. All MF metric-related analyses were performed with median-based dichotomization of cases into “metric-high” and “metric-low” groups.

As shown on Fig. 2A,B, both MF metrics were associated with shorter TTR as determined by Kaplan-Meier analyses and Cox regression analyses. ***f*****(α)**_**max**_ yielded the strongest results (HR = 2.5 (95% CI 1.50−4.18, p < 0.001)). Survival analyses with CSS yielded similar results (data not shown). High ***f*****(α)**_**max**_ was also associated with appearance of distant metastases and stage III tumors (Table 1).

For comparative purposes similar analyses were done using the histomorphological features. As shown in Fig. 2A, only the TBC was statistically significantly associated with TTR.

Multivariate analyses identified both MF metrics as independent prognostic factors for TTR (Table 3A,B). Among the histomorphological features only tumor border configuration remained statistically significant in the multivariate analyses (Supp. Table 3). Interestingly, when combined, the strongest histomorphological and MF predictor, both acted as independent prognostic factors (Table 3C).

Additional analyses were performed to test the prognostic value of MF metrics in a stage-dependent manner. As shown at Supp. Fig. 3, ***f*****(α)**_**max**_ preserved its prognostic strength in both groups while **α**_**max**_ acted as a strong prognostic factor in stage II, but not in stage III.

Together, these data identify MF metrics as independent prognostic factors, performing better than grade and budding, for recurrence in stage II-III colon cancer. ***f*****(α)**_**max**_ showed the strongest link with outcome and was also significantly associated with survival in both stage II and III.

### MF metrics define a patient group which benefit from adjuvant chemotherapy

We investigated if morphological tumor features, as defined by MF metrics or the traditional histomorphological characteristics, were associated with benefit of adjuvant chemotherapy. The effects of adjuvant chemotherapy on TTR were analyzed in subgroups of patients defined by their morphological features.

**Figure 3:**
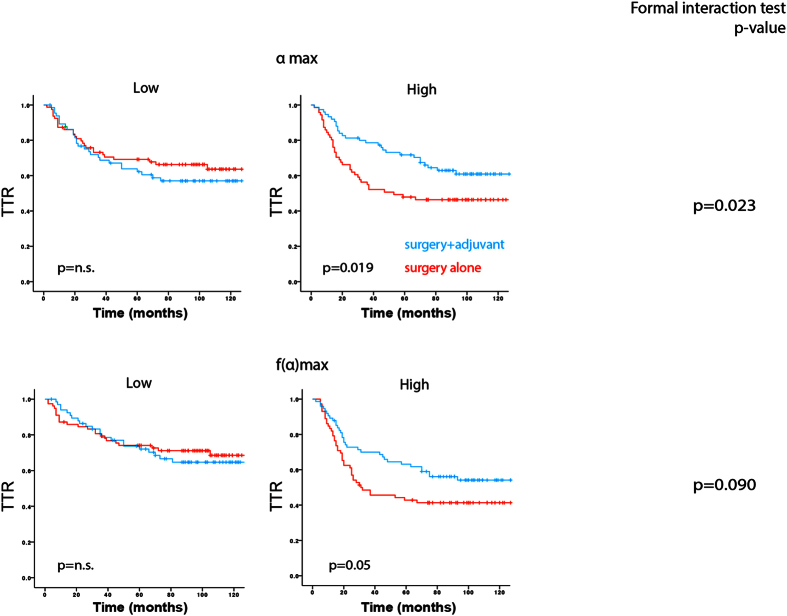
Treatment-efficacy in MF-metric-defined subgroups of stage II-III colon cancer. (Left and middle part) Kaplan-Meier plots illustrating TTR of stage II-III colon cancer patients receiving surgery alone (red lines) or surgery together with adjuvant chemotherapy after dichotomization of the study cohort based on **α**_**max**_ (upper part), or ***f*****(α)**_**max**_ (lower part). Log-rank test was used for statistical analyses. (Right part) Interaction between MF metrics and treatment were analyzed using “formal interaction test”. All MF metric-related analyses were based on median-based dichotomization of cases into “metric-high” and “metric-low” groups.

Interestingly, adjuvant treatment significantly improved TTR in the subgroups with high **α**_**max**_ but not in the low-score groups (Fig. 3, left and middle part). These findings were further supported by the interactions test, which demonstrated significant interactions between marker status and treatment in the high **α**_**max**_ group (Fig. 3, right part). The analyses of the images with included tumor internal structure fractal characteristics yielded similar results (Fig. S4).

Notably, none of the histomorphological scores showed any significant interaction with adjuvant treatment (Fig. S5).

## Discussion

The relationship between morphology and biological properties of tumors is well established and illustrated by the use of features such as grade, tumor border configuration (TBC) and tumor budding (TB) in colon cancer diagnosis. This study presents an alternative to these observer-dependent scoring methods by the use of multifractal analyses of scanned cytokeratin-stained tissue sections.

Multifractal analyses have been used in earlier studies to characterize different aspects of tumor morphology, including nuclear morphology, tissue architecture as determined by binarized H&E stainings and growth pattern/tumor-stroma interface identified by cytokeratin staining^12^,^13^,^14^,^17^,^23^,^24^. Some of these studies have also linked these metrics to outcome and have suggested relationships to intrinsic aggressiveness of disease or response to chemotherapy^12^,^24^. Notably, none of the earlier studies have relied on analyses of samples from randomized studies and analyses have therefore not been able to stringently distinguish between associations related to the natural course of the disease and response to treatment. The present study is thus the first study to stringently report on the ability of different MF metrics to act either as prognostic or response-predictive markers in cancer.

The present analysis demonstrates that MF metrics act as independent prognostic markers. Among the visually defined morphological characteristics, tumor differentiation grade is clinically implemented as a prognostic factor but still belongs to the category IIA prognostic factors^7^. TBC and TB have been extensively studied during past decades as prognostic factors. American Joint Committee on Cancer/UICC recommended the assessment of TBC^9^. However, the lack of established evaluation systems and poor reproducibility of the scoring have hampered practical implementation of TB and TBC^25^,^26^. Our finding that the MF metrics performs equally well, or better, than differentiation grade, TB and TBC as prognostic markers should prompt further validating studies on MF metrics as prognosticators. Multi-variable analyses including the MF metrics and the three visually defined features indicated that ***f*****(α)**_**max**_ and TBC acted as independent markers and thus possibly can be used together in future multiparametric risk scores.

This study also examined the potential ability of MF metrics to act as predictors of benefit of adjuvant 5-FU–based chemotherapy. Interestingly, median-based dichotomization of patients based on **α**_**max**_, or **α**_**max**_^**internal structure**^, identified patient groups which displayed differential benefit of treatment, in a manner where the high-marker-group showed significant improvement of CSS. Notably, none of the three visually defined histomorphological characteristics (tumor grade, TB and TBC) demonstrated any significant interactions with treatment.

Identification of markers predicting benefit of adjuvant chemotherapy is a highly active research area^1^. Some candidate markers subject to ongoing validation include thymidylate synthase and MSI^27^,^28^,^29^,^30^. Notably, significant interactions with treatment have not yet been demonstrated for any of these earlier candidate markers.

It is noted that the present study relies on a patient cohort where the chemotherapy regimen is different from the schedules currently in use. An important task for future studies will therefore be to validate the indications of response-predictive potential of MF metrics in other, more modern, tumor collections. However, none of these modern collections will provide an opportunity to analyze the effects on a surgery-alone group. Even if the absolute risks of recurrence have diminished due to improvements with time from the 1990s, the relative importance of the MF metrics reported here is likely robust.

Future studies should also aim to define the biology, which is captured by the MF metrics. Such studies can possibly be done by collecting MF metrics from tumor collections, which are well-annotated with regard to molecular features such as gene-expression patterns and mutation profiles.

Digital pathology and whole slide imaging is presently becoming part of routine at many pathology departments. This development is encouraging for the present study since it is lowering the technological threshold for implementation of MF metrics as routine biomarkers. Findings from the present study should also encourage other independent efforts to fully exploit the potential of digital image analyses for detection of clinically relevant biology impacting on prognosis and response to treatment.

## Additional Information

**How to cite this article**: Mezheyeuski, A. *et al.* Image analysis-derived metrics of histomorphological complexity predicts prognosis and treatment response in stage II-III colon cancer. *Sci. Rep.* **6**, 36149; doi: 10.1038/srep36149 (2016).

**Publisher’s note:** Springer Nature remains neutral with regard to jurisdictional claims in published maps and institutional affiliations.

## Supplementary Material

Supplementary Information

## Figures and Tables

**Table 1:** Associations between MF metrics and clinicopathological characteristics.

Characteristic	n	Outline multifractal metrics			
		α _max_	p	f(α) _max_	p
Age (Years)					
<66	130	1.696	n.s.	1.541	n.s.
≥66	161	1.697		1.547	
Sex					
Male	146	1.676	0.027	1.523	0.008
Female	145	1.717		1.566	
Tumor Site					
Proximal^a^	154	1.732	<0.001	1.573	<0.001
Distal colon	137	1.657		1.512	
Mismatch repair status					
MMR proficient	227	1.692	n.s.	1.544	n.s.
MMR deficient	56	1.711		1.554	
Missing data	7				
Stage					
II	134	1.691	n.s.	1.520	0.001
III	157	1.701		1.565	
Adjuvant Chemotherapy					
Yes	141	1.698	n.s.	1.544	n.s.
No	150	1.695		1.545	
Local Recurrence					
With	25	1.701	n.s.	1.587	n.s.
Without	266	1.696		1.540	
Distant Metastases					
With	79	1.720	n.s.	1.573	0.008
Without	212	1.688		1.533	

Abbreviations: n, number of cases; p, p-value; n.s., not statistically significant. Mann–Whitney U test was used.

^a^To splenic flexure.

**Table 2:** Associations between MF metrics and histomorphological characteristics.

Characteristic	n	Outline multifractal metrics			
		α _max_	p	f(α) _max_	p
Tumor border configuration					
Pushing	88	1.649	<0.001**	1.483	<0.001**
Intermediate	70	1.704		1.535	
Infiltrative	131	1.725		1.591	
Missing data	2				
Budding					
Low	198	1.680	0.001*	1.513	<0.001*
High	91	1.733		1.591	
Grade of Differentiation					
Well (G1)	24	1.702	n.s.**	1.536	0.017**
Moderate (G2)	200	1.686		1.533	
Poor (G3)	54	1.727		1.583	
Missing data	3				

Abbreviations: n, number of cases; p, p-value; n.s., not statistically significant. Mann–Whitney U test* and Kruskal-Wallis statistical test** were used.

**Table 3:** Multivariable analyses of α _max_ (A) and f(α)_max_ (B) together with standard clinical characteristics as prognostic factors for time to tumor recurrence in surgery-alone-treated stage II-III colon cancer patients.

	Covariates	HR	95.0% CI for HR		p-value
			Lower	Upper	
A	**α**_**max**_	1.947	1.147	3.307	0.014
	Stage (III vs II)	2.241	1.290	3.893	0.004
	Gender (male vs female)	1.314	0.785	2.201	0.299
	Age > = 66	1.268	0.753	2.132	0.372
	MMR status (proficient vs deficient)	1.552	0.712	3.383	0.269
	Localization (proximal vs. distal)	1.102	0.641	1.895	0.725
B	**f(α)**_**max**_	2.541	1.448	4.460	0.001
	Stage (III vs II)	1.912	1.087	3.362	0.024
	Gender (male vs female)	1.236	0.740	2.063	0.418
	Age > = 66	1.110	0.659	1.870	0.695
	MMR status (proficient vs deficient)	1.492	0.686	3.248	0.313
	Localization (proximal vs. distal)	1.175	0.683	2.021	0.561
C	**f(α)**_**max**_	2.068	1.144	3.740	0.016
	**Tumor border configuration**	2.143	1.233	3.724	0.007
	Stage (III vs II)	1.902	1.086	3.332	0.025
	Gender (male vs female)	1.297	0.771	2.180	0.327
	Age > = 66	1.087	0.643	1.836	0.756
	MMR status (proficient vs deficient)	1.282	0.586	2.803	0.534
	Localization (proximal vs. distal)	1.257	0.729	2.168	0.411

(C) Multivariable analysis which includes the strongest visual and digital predictors (tumor border configuration and f(α)_max_).

Abbreviations: HR, hazard ratio; CI, confidence interval.
