# Supplementary material for: Image analysis-derived metrics of histomorphological complexity predicts prognosis and treatment response in stage II-III colon cancer
Source: Sci Rep. 2016 Nov 2;6:36149. doi: 10.1038/srep36149 (PMC5095346; doi:10.1038/srep36149)

Supplementary materials

Image analysis-derived metrics of histo-morphological complexity predicts prognosis and treatment response in stage II-III colon cancer

Authors: Artur Mezheyeuski[1,2], Ina Hrynchyk[3], Mia Karberg[1], Anna Portyanko[2], Lars Egevad[1], Peter Ragnhammar[1], David Edler[4], Bengt Glimelius[5], Arne Östman[1]

Affiliations:

1.Department of Oncology-Pathology, Karolinska Institutet, Stockholm, Sweden;

2.Department of Pathology, Belarusian State Medical University, Minsk, Belarus;

3.City Clinical Pathologoanatomic Bureau, Minsk, Belarus

4.Department of Molecular Medicine and Surgery, Karolinska University Hospital Solna, Stockholm, Sweden

5.Department of Immunology, Genetics and Pathology, Uppsala University, Uppsala, Sweden;

Fig. S1.

Image analyses pipeline. (A) Digital images of the tumor sections stained with pan-cytokeratin (brown) and haematoxylin (blue). (B) Modified images with segmented cancer tissue (red). (C) Tumor external contours outlined (red) (C1) or tumor external contours and contours of internal tumor structures outlined (red) (C2), reflecting the tumor morphological organization. (D) Multifractal analysis with FracLac. (E) Summary plot showing combined case-derived data from *D(q) vs Q* spectra. Wickers indicate standard errors.

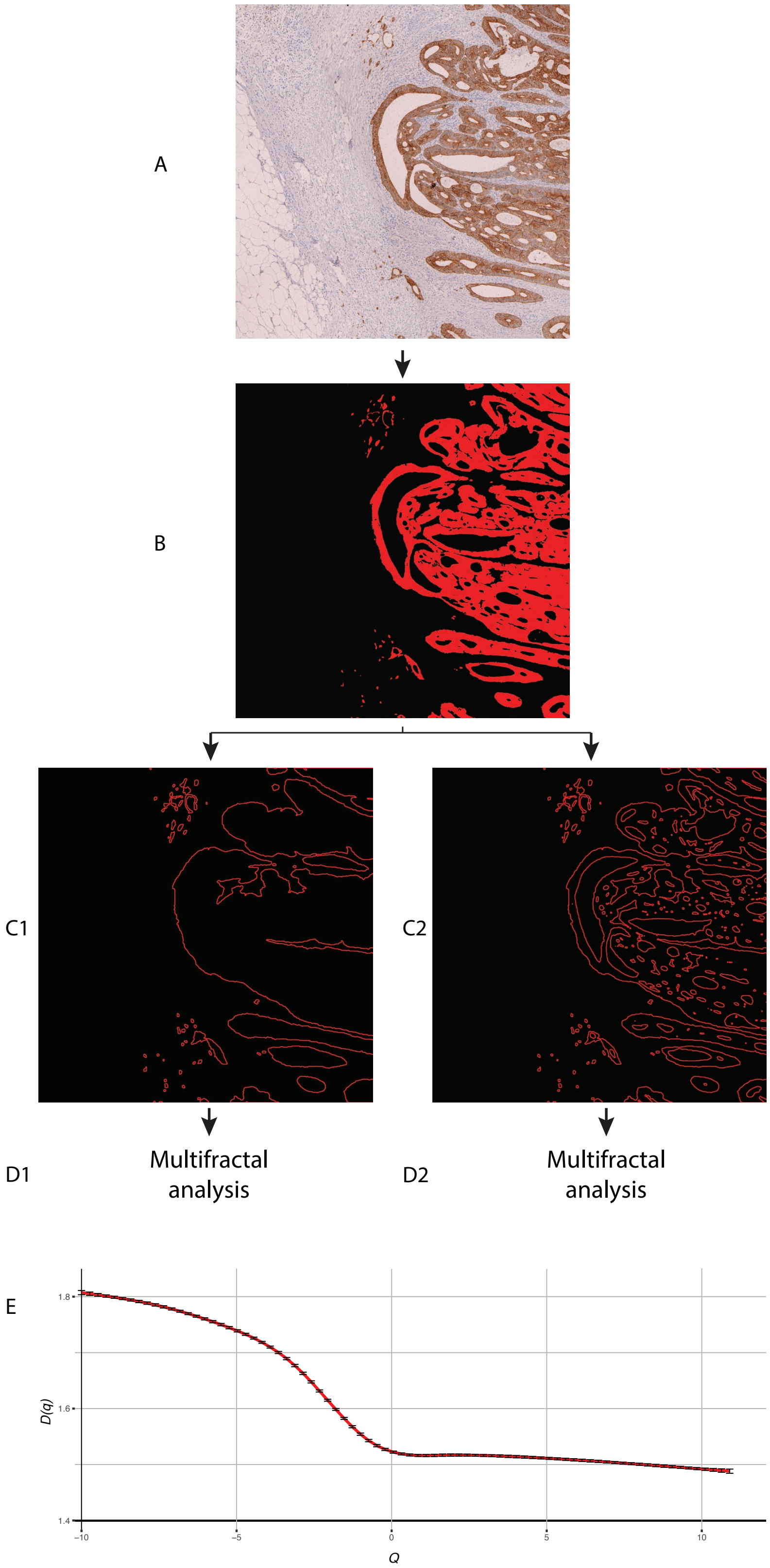
A
B
C1
C2
D1
Multifractal analysis
D2
Multifractal analysis
E
D(q)
Q
1.8
1.6
1.4
−10
−5
0
5
10

Table S1.

Associations between MF metrics and clinicopathological characteristics. The MF metrics derived from the analyzes of tumor internal structure are used

		Structural multifractal metrics			
Characteristic	n	**α_{max} internal structure**	p	**$f(\alpha)_{max}$ internal structure**	p
**Age (Years)**					
< 66	130	1.789	n.s.	1.658	n.s.
≥ 66	161	1.808		1.674	
**Sex**					
Male	146	1.794	n.s.	1.653	n.s.
Female	145	1.804		1.681	
**Tumor Site**					
Proximal	154	1.818	0.003	1.688	0.001
Distal colon	137	1.778		1.643	
**Mismatch repair status**					
MMR proficient	227	1.791	n.s.	1.666	n.s.
MMR deficient	56	1.823		1.676	
**Stage**					
II	134	1.799	n.s.	1.654	n.s.
III	157	1.799		1.678	
**Adjuvant Chemotherapy**					
Yes	141	1.801	n.s.	1.675	n.s.
No	150	1.797		1.658	
**Local Recurrence**					
With	25	1.809	n.s.	1.694	n.s.
Without	266	1.798		1.664	
**Distant Metastases**					
With	79	1.811	n.s.	1.677	n.s.
Without	212	1.794		1.663	

Abbreviations: n, number of cases; p, p-value; n.s., not statistically significant. Mann–Whitney U test was used

Table S2.

Associations between fractal metrics and histo-morphological characteristics. The MF metrics derived from the analyzes of tumor internal structure are used**.**

		Structural multifractal metrics			
Characteristic	n	**α_{max} internal structure**	p	**$f(\alpha)_{max}$ internal structure**	p
**Tumor border configuration**					
Pushing	88	1.793	n.s.	1.634	0.011
Intermediate	70	1.816		1.675	
Infiltrative	131	1.795		1.687	
**Budding**					
Low	198	1.787	0.015	1.649	<0.001
High	91	1.826		1.708	
**Grade of Differentiation**					
Well (G1)	24	1.756	n.s.	1.642	n.s.
Moderate (G2)	200	1.803		1.663	
Poor (G3)	54	1.802		1.687	

Abbreviations: n, number of cases; p, p-value; n.s., not statistically significant. Mann–Whitney U test and Kruskal-Wallis statistical tests were used.

Fig. S2.

Associations between adjuvant chemotherapy and CSS or TTR in stage II-III colon cancer patients in the studied cohort. Log-rank test used for statistical analyses.

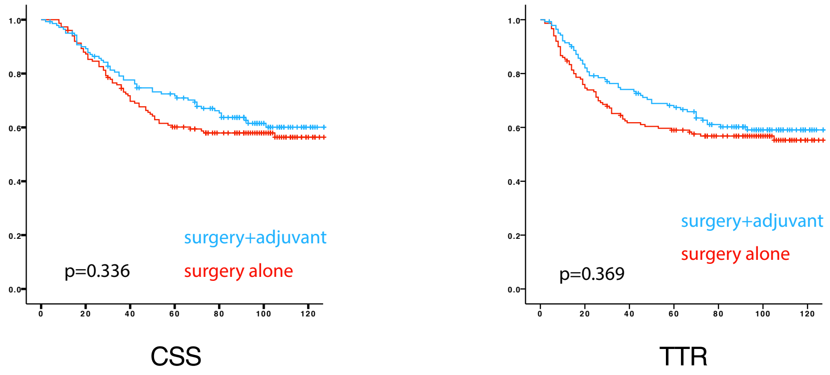

Table S3.

Multi-variable analyses of histo-morphological features together with standard clinical characteristics as prognostic factors for time to recurrence in surgery-alone-treated stage II-III colon cancer patients.

Covariates	HR	95.0% CI for HR		p-value
		Lower	Upper	
**Grade of Differentiation**	1.493	.800	2.787	.208
Stage (III vs II)	2.318	1.285	4.184	.005
Gender (male vs female)	1.412	.833	2.392	.200
Age >=66	1.329	.774	2.281	.302
MMR status (proficient vs deficient)	1.500	.675	3.332	.319
Localization (proximal vs. distal)	1.061	.607	1.855	.834

Covariates	HR	95.0% CI for HR		p-value
		Lower	Upper	
**Tumor border configuration**	2.637	1.550	4.489	.000
Stage (III vs II)	2.188	1.265	3.787	.005
Gender (male vs female)	1.407	.838	2.363	.196
Age >=66	1.195	.709	2.016	.503
MMR status (proficient vs deficient)	1.259	.571	2.776	.568
Localization (proximal vs. distal)	1.085	.637	1.849	.763

Covariates	HR	95.0% CI for HR		p-value
		Lower	Upper	
**Budding**	1.294	.755	2.217	.349
Stage (III vs II)	2.174	1.232	3.837	.007
Gender (male vs female)	1.259	.757	2.094	.375
Age >=66	1.191	.705	2.012	.514
MMR status (proficient vs deficient)	1.429	.653	3.128	.372
Localization (proximal vs. distal)	.965	.568	1.641	.896

Abbreviations: HR, hazard ratio; CI, confidence interval

Fig. S3.

Survival-associations for fractal metrics and histo-morphological scores in stage II and III colon cancer treated with surgery alone. Association between MF metrics (α_{max} and $f(\alpha)_{max}$), histo-morphological features and time to recurrence. Cox regression analyses were used for determination of HRs. All MF related analyses were based on median-based dichotomization of cases into "metric-high" and "metric-low" groups.

patients with surgery alone,
TTR

	metric	HR (95% CI)	p value
Stage II	α max	3.64 (1.41–9.42)	0.008
	f(α) max	2.64 (1.11–6.29)	0.028
	Diff. grade	0.81 (0.19–3.52)	n.s.
	TBC	4.11 (1.65–10.25)	0.002
	TB	1.44 (0.53–3.93)	n.s.
Stage III	α max	1.24 (0.69–2.25)	n.s.
	f(α) max	2.08 (1.09–3.94)	0.025
	Diff. grade	1.29 (0.69–2.41)	n.s.
	TBC	1.87 (1.02–3.41)	0.042
	TB	1.34 (0.74–2.42)	n.s.

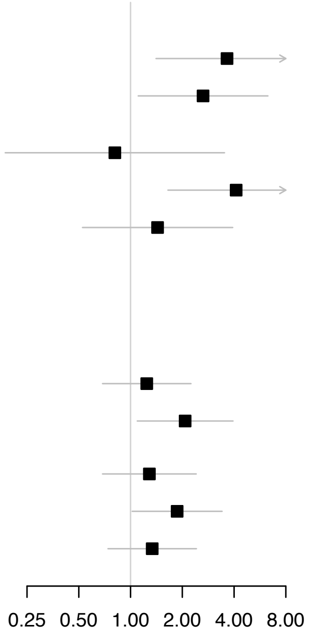

Fig. S4.

Treatment-efficacy in MF-metric-defined sub-groups of stages II-III colon cancer. (Left and middle part) Kaplan-Meier plot illustrating time to recurrence of stage II-III colon cancer patients receiving surgery alone (red lines) or surgery together with adjuvant chemotherapy after dichotomization of the study cohort based on $\boldsymbol{\alpha}_{\mathbf{max}}^{\mathbf{internal\ structure}}$ (upper part), $\boldsymbol{f(\alpha)}_{\mathbf{max}}^{\mathbf{internal\ structure}}$ (lower part). Log-rank test were used for statistical analyses. (Right part) Potential interaction between fractal metrics and treatment were analysed using "formal interaction test". All MF metric-related analyses were based on median-based dichotomization of cases into "metric-high" and "metric-low" groups.

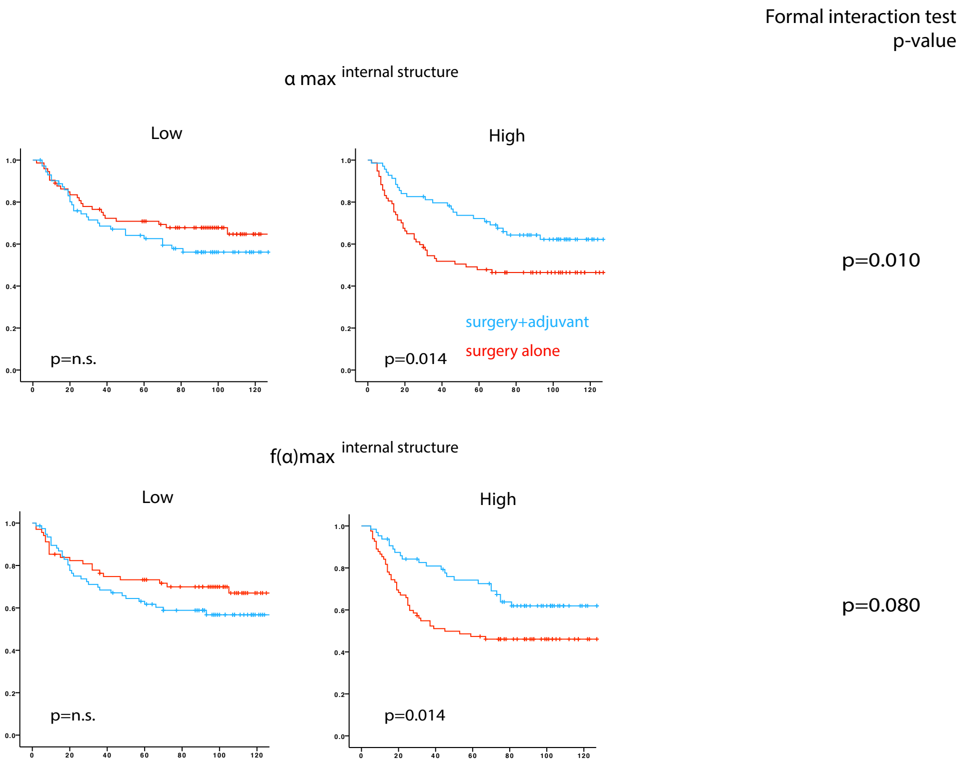

Fig. S5.

Treatment-efficacy in histo-morphology-defined sub-groups of stages II-III colon cancer. (Left and middle part) Kaplan-Meier plots illustrating time to recurrence of stage II-III colon cancer patients receiving surgery alone (red lines) or surgery with adjuvant chemotherapy after dichotomization of the study cohort based on tumor differentiation (upper part), tumor border configuration (middle part), budding (lower part). Log-rank test were used for statistical analyses. (Right part) Potential interaction between fractal metrics and treatment were analyzed using "formal interaction test".

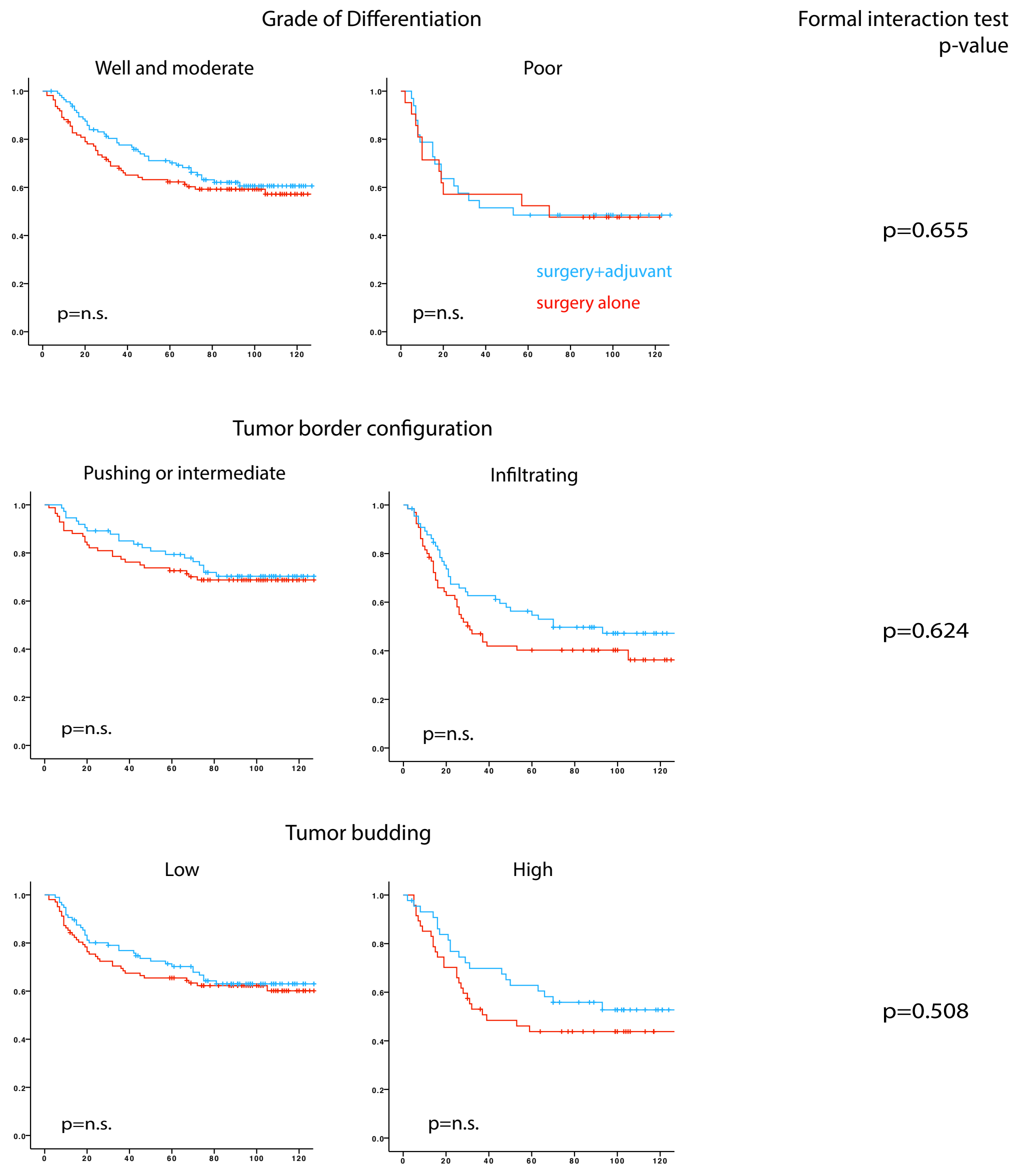

Supplement: Supplementary Information [file srep36149-s1.pdf]
